# Empirical Bayes estimation of the selected treatment mean for two-stage drop-the-loser trials: a meta-analytic approach

**DOI:** 10.1002/sim.5920

**Published:** 2014-07-22

**Authors:** Jack Bowden, Werner Brannath, Ekkehard Glimm

**Affiliations:** a MRC Biostatistics Unit Hub for Trials Methodology ResearchCambridge, U.K.; b Competence Center for Clinical Trials Bremen, Faculty 3, University of BremenBremen, Germany; c Novartis Pharma AGCH-4002 Basel, Switzerland

**Keywords:** drop-the-loser trials, empirical Bayes estimation, meta-analysis, temporal coherency

## Abstract

Point estimation for the selected treatment in a two-stage drop-the-loser trial is not straightforward because a substantial bias can be induced in the standard maximum likelihood estimate (MLE) through the first stage selection process. Research has generally focused on alternative estimation strategies that apply a bias correction to the MLE; however, such estimators can have a large mean squared error. Carreras and Brannath (*Stat. Med.* 32:1677-90) have recently proposed using a special form of shrinkage estimation in this context. Given certain assumptions, their estimator is shown to dominate the MLE in terms of mean squared error loss, which provides a very powerful argument for its use in practice. In this paper, we suggest the use of a more general form of shrinkage estimation in drop-the-loser trials that has parallels with model fitting in the area of meta-analysis. Several estimators are identified and are shown to perform favourably to Carreras and Brannath's original estimator and the MLE. However, they necessitate either explicit estimation of an additional parameter measuring the heterogeneity between treatment effects or a quite unnatural prior distribution for the treatment effects that can only be specified after the first stage data has been observed. Shrinkage methods are a powerful tool for accurately quantifying treatment effects in multi-arm clinical trials, and further research is needed to understand how to maximise their utility.

## 1. Introduction

Two-stage drop-the-loser designs provide a framework for picking the most effective treatment out of a larger group of candidates and then testing it against a standard therapy in a confirmatory analysis. Although this design is an efficient way to discover effective treatments, the selection mechanism acts to inflate the type I error of the final test statistic 1,2 and can also induce a substantial bias into the standard maximum likelihood estimate (MLE). With regard to the former, current regulatory authority guidance (e.g. 3) is unequivocal that the final analysis must control the type I error rate. With regard to the latter, whilst acknowledging that estimation bias is a serious issue affecting the validity of adaptive trials and that bias should be ‘minimised’, there is a distinct lack of guidance and consensus on how this should be achieved. Research has generally focused on estimators that apply a bias correction to the MLE. One such class of estimators, referred to as uniform minimum variance conditionally unbiased estimators (UMVCUEs), totally removes the MLE's bias 4–7. Others have proposed iterative or likelihood-based methods that can substantially reduce the bias of the MLE, without being unbiased 8,9,7. Unfortunately, methods that explicitly target bias correction generally lead to an estimator with a mean squared error (MSE) larger than that of the MLE.

Carreras and Brannath 10 have recently proposed the use of shrinkage estimation 11 within the context of a two-stage drop-the-loser trial. In their method, the stage 1 data on all treatments are used to define a shrinkage estimate for the selected treatment at stage 1. This is then combined with the stage 2 estimate for the selected treatment via a weighted average. Under the assumption that the true treatment effects are independent and follow a common normal distribution (with any mean and variance), their estimator is shown to dominate the MLE in terms of MSE, thus providing a very powerful argument for its use in practice. In this paper, we propose an alternative shrinkage estimation strategy for drop-the-loser designs. Our approach is, in some ways, a simpler estimation procedure to that of 10 because it uses all of the available data within a single, standard, shrinkage equation. However, this apparent simplicity does impose some additional complications, which are discussed at length herein.

In Section 2, we introduce our notation for the two-stage drop-the-loser design. In Section 3, we describe the general principle of shrinkage estimation, Carreras and Brannath's original application of shrinkage estimation to the drop-the-loser trial context, and also present our alternative approach. In Section 4, we introduce several shrinkage estimators that naturally flow from our alternative formulation, and in Section 5, we evaluate the performance of the existing and alternative shrinkage estimators for various two-stage drop-the-loser design scenarios. We conclude in Section 6 with a discussion of the issues raised and point to further avenues of research.

## 2. The two-stage drop-the-loser design

Let 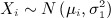, *i* = 1, … ,*k*, be the effect estimates (MLEs) of *k* experimental treatments *T*_1_,…,*T*_*k*_ at the first stage of a two-stage trial. The common variance term, 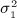, is assumed to be known. Assuming that large values indicate the most benefit, the ‘best’ treatment, *T*_*s*_, *s* ∈ {1,…*k*}, is selected as the one with the top-ranking MLE. That is, *X*_*s*_ = Max {*X*_1_,…,*X*_*k*_}. Treatment *T*_*s*_ is taken forward in isolation for testing on an independent population in stage 2. Let *Y*_*s*_ 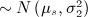 be the estimate for *μ*_*s*_ at stage 2.

Let *X*_0_ and *Y*_0_ represent the normally distributed treatment effect estimates for the control group at stages 1 and 2, with mean *μ*_0_ and variances 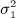 and 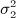, respectively. At the end of the trial, we are interested in estimating the contrast *μ*_*s*_ − *μ*_0_. Because the control group always proceeds to the final stage, *μ*_0_ is unbiasedly estimated by its MLE, and we therefore focus our attention on estimation of *μ*_*s*_ only. The MLE of *μ*_*s*_ at stage 2 and its (assumed) variance are given by 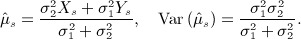
1 Because it ignores the selection of *X*_*s*_, 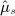 is positively biased (potentially seriously so), and 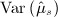 is also incorrect. We can express the most efficient unbiased estimate for *μ*_*s*_ as 
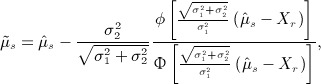
2 where *ϕ*(.) and Φ(.) are the standard normal density and distribution functions, respectively, and where *X*_*r*_ is defined as the second best performing treatment at stage 2, that is, *X*_*r*_ = Max {*X*_1_,…,*X*_*k*_} / *X*_*s*_. This is referred to as the UMVCUE 4,6.

### 2.1. Assessing estimators of *μ*_*s*_

For any estimator of *μ*_*s*_, 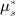, we can express its bias and MSE as 
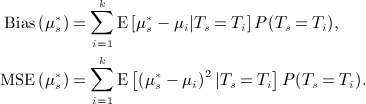
3 This form (from Posch 12) makes clear that at the trial outset, *μ*_*s*_ is a random variable. By definition, 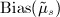 = 0, and 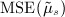 is smaller than any other 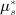 that is also unbiased. This of course does not mean that 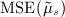 is smaller than any biased estimator; for example, it is generally true that 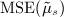 ≫ 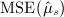 (see Section 5 for example). Furthermore, it is commonly agreed that MSE (a measure equivalent to its variance + squared bias) provides a far better summary of an estimator's worth than bias alone.

## 3. Shrinkage estimation

The standard motivation for using shrinkage methods is to provide simultaneous, accurate estimation for a group of parameters, where accuracy is defined via the combined MSE. For example, if we were interested in jointly estimating the true mean effect of all *k* treatments *μ*_1_,…,*μ*_*k*_ using only stage 1 data, then it will generally be true that the combined MSE, 
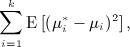
4 is far smaller when 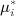 equals 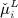 as opposed to the MLE *X*_*i*_, where 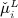 is Lindley's estimator 13: 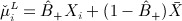
5 and 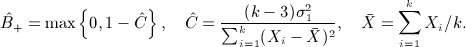
6 Although shrinkage formula (5) was not originally proposed using a Bayesian argument, it can be easily understood and shown to be optimal within a Bayesian framework. Assume that *a priori μ*_1_,…,*μ*_*k*_ are themselves independent and identically distributed (i.i.d) *N*(*μ*,*τ*^2^) random variables and only stage 1 data are available for the *k* treatments. Given *μ*_*i*_, the distribution of its MLE *X*_*i*_ is 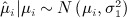. The *posterior* distribution of *μ*_*i*_ given 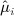 is then 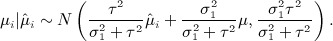
7

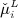
 can therefore be viewed as an ‘Empirical Bayes’ estimate for the posterior mean of equation (7), with 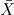, *X*_*i*_ and 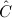 substituted for *μ*, 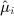 and 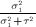, respectively. 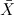 is clearly an unbiased estimate of *μ*, but it is perhaps less obvious that 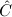 is an unbiased estimate for 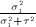, regardless of the true value of *τ*^2^. If 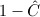 gives a negative value, it is replaced by 0 in the definition of 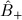. This ‘plus rule’ has been shown to further reduce the MSE of the resulting estimate 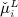 14.

### 3.1. Carreras and Brannath's approach

Hwang 15 explicitly considered estimation of a single mean parameter from a *k* component system, where all *k* components have normally distributed estimates with a common variance and the single component is identified by having the largest estimate. This is identical to estimating *μ*_*s*_ using only stage 1 data in a two-stage drop-the-losers trial. He proved that when the treatment means follow a *N*(*μ*,*τ*^2^) prior distribution (for any *μ* and 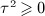), so that their posterior distributions obey equation (7) and 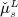 is defined by equation (5) with *i* = *s* (i.e. drop-the-losers selection), then the dominance result 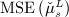 *≤* MSE(*X*_*s*_) holds. Within the context of a two-stage drop-the-loser trial, Carreras and Brannath use Hwang's result to show that their estimator for *μ*_*s*_ at stage two 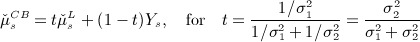
8 analogously dominates 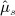 from equation (1). Their result relies on the fact that equation (1) is equivalent to replacing 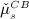 in equation (8) with *X*_*s*_. This also occurs naturally when the shrinkage factor *C* is set to 0.

### 3.2. An alternative formulation

Carreras and Brannath's method for estimating *μ*_*s*_ following a two-stage drop-the-loser trial can itself be viewed as a two-stage approach. That is, only the stage 1 data are used in the standard shrinkage estimator 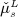, and then, stage 2 data on the selected treatment, *Y*_*s*_, are added separately in equation (8) afterwards. Although 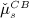 has the nice dominance property over the MLE, it is useful to consider whether it can itself be improved upon. For example, why not use all of the stages 1 and 2 data to define a single shrinkage estimator? Although this sounds straightforward, it does impose some extra complications. Despite being only truly concerned with estimation of the top-ranking treatment's mean, *μ*_*s*_, Carreras and Brannath's method is defined to find shrinkage estimates given *k* parameter estimates with a *common* variance. This means that we are assuming the posterior distribution for *μ*_*i*_ implied by (7) (ignoring the stage 2 data), which enables the use of 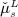 from (5). However, if we use all of the data (including the stage 2 data *Y*_*s*_), we may assume the following set-up: 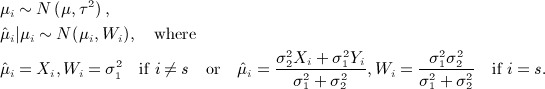


Further assuming that the *μ*_*i*_s are stochastically independent and the 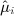
s are conditionally independent given *μ*_*i*_, then 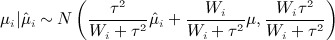
9 becomes the distribution with which to construct shrinkage estimates for the *μ*_*i*_s. The fact that *W*_*i*_ is not constant makes specification of single appropriate shrinkage factor *C* and estimate for the grand mean *μ* far less straightforward. In Section 4, we will discuss estimation for this new setting, pointing out its connection with model fitting in the area of meta-analysis.

## 4. Estimation for the new target

### 4.1. Direct estimation

Under the framework of equation (9), if the parameters *μ* and *τ*^2^ were known, the best estimate for *μ*_*s*_ is given by 
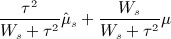
10 Furthermore, with infinite data, it is clear that directly replacing *μ* and *τ*^2^ in this expression with consistent estimates, 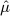 and 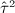, would give equation (10). If one assumes that *τ*^2^ is known, then the variance of the posterior distribution in (9) also becomes completely known. It is then possible to show that the MLE for *μ* is 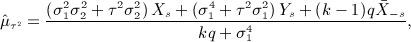
11 where 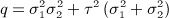 and 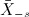 = 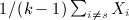. *τ*^2^ could then be estimated by maximising the posterior likelihood of (9) with respect to *τ*^2^ at 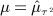. See the Appendix for further details. We will refer to the estimate for *μ*_*s*_ obtained by directly plugging in the previous estimates to equation (10) as 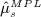. Although this approach is fairly crude, it will be interesting to observe the performance of 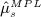 compared to several ‘legitimate’ shrinkage estimators that are now introduced.

### 4.2. Shrinkage estimation: Carter and Rolph's standard prior approach

Carter and Rolph 16 investigate shrinkage methods for jointly estimating the parameters of a *k* component system with unequal variances, as set up in equation (9). We will use it specifically to yield an estimate for *μ*_*s*_ in the context of a two-stage drop-the-loser trial of the form 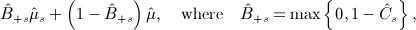
12 so as to approximate equation (10). Although not immediately obvious, it can be shown that their suggested approach is equivalent the following procedure. First define a new weight *V*_*i*_ = (*W*_*i*_ + *τ*^2^), and find the value of *τ*^2^ that solves *Q*(*τ*^2^) = *k* − 1, where 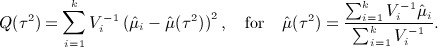
13 If *Q*(*τ*^2^) = *k* − 1 for a *τ*^2^ < 0, then the estimate is truncated to 0. *Q*(*τ*^2^) is known in the area of medical meta-analysis as the ‘generalised’ Q statistic 17,18, and this estimation method for *μ* is known as the Paule–Mandel method of moments algorithm 19,20. Let the estimate for *τ*^2^ derived in this manner be equal to 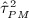. The shrinkage factor for *μ*_*s*_ is then approximated by 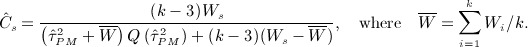
14 The form of 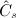 may appear complicated, but it has the nice property that if the *W*_*i*_s are all equal, then it reduces to the original 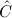 given in equation (6). In our context, the *W*_*i*_s can only approach equality as 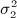 → ∞ or as the stage 2 sample size tends to zero. 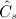 can be inserted into equation (12) along with the MLE 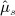 and grand mean 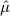 given by 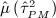, to yield a new shrinkage estimator for *μ*_*s*_. We will refer to this estimator as 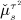 – the ‘ *τ*^2^’ denoting that this parameter is additionally and explicitly estimated.

### 4.3. Shrinkage estimation: Carter and Rolph's proportional prior approach

Carter and Rolph 16 also propose an alternative method for applying shrinkage estimation within the unequal variance context, which usefully avoids an iterative estimation of *τ*^2^. It relies on the assumption of a different prior distribution for the treatment parameters, namely *μ*_*i*_ ∼ *N*(*μ*,*W*_*i*_*τ*^2^). This asserts that the prior uncertainty around each treatment's mean is directly proportional to the variance of its estimate, 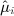. By replacing *τ*^2^ with *W*_*i*_*τ*^2^ in (9), it is clear that this implies the posterior distribution: 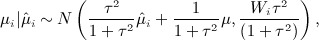
15 the mean of which becomes an alternative target to estimate. Because this mean does not depend on *W*_*i*_, it suffices to calculate a single shrinkage factor 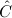 using all of the data. This can then, in conjunction with an estimate for *μ*, be used to estimate *μ*_*s*_ via equation (12). Turning first to estimation of *μ*: Under the proportional prior, the unconditional distribution of the estimates is 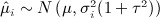. Therefore, given weights 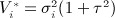, the inverse variance weighted average 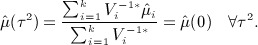


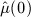
 is referred to in meta-analysis as the ‘fixed-effects’ estimate for *μ*, as opposed to a ‘random-effects’ estimate, of which 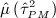 is an example. Of course, when *τ*^2^ is estimated to be 0, they are equal.

Turning now to estimation of *μ*_*s*_ via 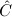:It can easily be shown that Carter and Rolph's approach in this context is equivalent to choosing: 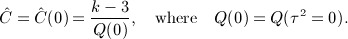
16

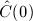
 can be seen as a simple generalisation of the 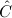 in equation (6) for the case where the *W*_*i*_ terms are not constant. *Q*(0) is known as Cochrane's heterogeneity statistic in meta-analysis and is closely related to the DerSimonian and Laird estimator for *τ*^2^, 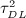 20. For example, when *Q*(0) > *k* − 1, 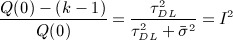


where 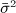 is called the ‘typical’ within study variance and *I*^2^ is a popular measure of ‘inconsistency’ (heterogeneity) among studies in a meta-analysis 21. We will refer to the resulting estimator (which utilises 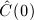 and 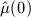) as 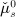.

### 4.4. Incorporating ‘Limiting Translation’

It is well known that shrinkage methods can perform poorly with respect to specific parameter components of a larger system, when the magnitude of the specific parameters are among the most extreme. For this reason, Efron and Morris 22 suggest the use of a ‘Limiting Translation’ (LT) strategy that constrains one shrinkage estimator to be within a certain distance of the MLE. Efron 14 and Johnson 23 suggest a practical choice for this constraint of one unit of the MLE's standard error. The effect of applying LT to a single extreme parameter component of a larger system, say a *μ*_*i*_ from *μ*_1_,…,*μ*_*k*_, is to dramatically reduce the *i*’th contribution to the overall MSE of equation (4), at the expense of increasing the total value of equation (4) by a small margin.

Although from equation (3), we can see that, at the trial outset, *μ*_*s*_ is not a single parameter but rather a weighted mixture of all *k* fixed parameter values *μ*_1_,…,*μ*_*k*_, the specific values of those parameters may mean that *μ*_*s*_ is consistently an outlier. For example, this would certainly be the case if one treatment was far more effective than any other because it would monopolise the value of *μ*_*s*_. We can apply LT to the shrinkage estimator 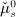 by subtracting 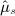 from equation (12), constraining the result to be 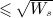 and noting that the definition of 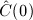 in equation (16) becomes 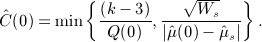
17 This estimator will be referred to as 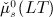. LT versions of all other estimators are clearly possible but are not considered here.

### 4.5. Some implications of using 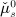


When deriving the form of 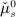, there is no inherent mathematical difficulty in assuming an *N*(*μ*,*W*_*i*_*τ*^2^) prior distribution (with varying *W*_*i*_s) for the *μ*_*i*_s because the resulting posterior distribution for 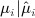 remains in the normal family. However, this shrinkage approach does raise certain philosophical questions when applied in the context of a two-stage drop-the-loser trial. The primary issue is that we do not know *a priori* which treatment will be selected. So, assigning the *N*(*μ*,*W*_*s*_*τ*^2^) prior to *μ*_*s*_ (for general values of *μ* and *τ*^2^) is only possible after we have observed the first stage data. This violates the principle of ‘Temporal Coherency’ 24 that states that the prior must be specified in advance and constant in time. Indeed, this principle is overwhelmingly adhered to by practitioners of Bayesian inference in the interest of maintaining scientific objectivity. A consequence of this temporal violation, which becomes most apparent in Section 5, is that there is no general way to simulate data consistent with the assumptions of 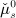. To understand this, suppose we wanted to generate trial data consistent with the shrinkage estimator 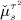 instead. We simply start by simulating the *μ*_*i*_s from an *N*(*μ*,*τ*^2^) density given values for *μ* and *τ*^2^, which can then be used to generate the trial data for stages one and two (*X*_1_,…,*X*_*k*_,*Y*_*s*_). These data can then be used to specify the distributions 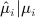 and 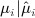 from equation (9). Clearly, we can not follow an equivalent data-generating procedure when the *μ*_*i*_s come from an *N*(*μ*,*W*_*i*_*τ*^2^) prior density because, as previously stated, the prior can only be specified *after* seeing the data. The single exception is when *τ*^2^=0 (implying a degenerate normal prior) in which case the *μ*_*i*_s all take the value *μ* with probability 1. This is equivalent to assuming a fixed effects model with only one unknown parameter, *μ*.

Of course, despite these philosophical concerns, we are still free and able to evaluate 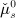 in a simulation study without exactly mimicking the data-generating process it relies upon.

## 5. Simulation study

We simulate trial data under a two-stage drop-the-loser design in order to quantify the bias and MSE of four new estimators 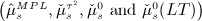 for *μ*_*s*_, alongside the existing estimators 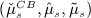. We use the definition of bias and MSE from equation (3), which can be simply and accurately approximated by averaging over all simulations where, in each single case, a treatment *T*_*i*_ out of *k* is ranked top at the end of stage 1 so that *μ*_*s*_ = *μ*_*i*_. We chose four different levels of standard error associated with the stage 1 and 2 estimates, along with four different scenarios for the six unknown means:

- Scenario *I*. True means ∼ *N*(0,1)
- Scenario *II*: True means all 0
- Scenario *III*: One true mean = 1, 5 means equal to 0
- Scenario *IV*: One trgue mean = 1.5, 5 means equal to 0.

The underlying distribution of the treatment parameters is a key factor driving the Bayesian motivation of any shrinkage estimator. Scenario *I* is compatible with the prior assumptions of 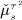 and 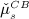. Scenario *II* is compatible with the prior assumptions of all shrinkage estimators, despite being non-stochastic, because it is equivalent to scenario *I* with *τ*^2^ = 0. Carreras and Brannath's dominance result for 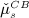 is valid for scenarios *I* and *II*. Scenarios *III* and *IV* are not compatible with any shrinkage estimator. However, all scenarios are compatible with the assumptions of the UMVCUE, in the sense that it maintains its unbiasedness for any constellation of parameter values.

We show the results for the 16 simulation scenarios in Table1. All reported figures are based on 50000 simulations. For each simulation scenario, we show the bias and 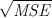 in units of the MLE 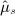
s naive standard error, 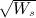, to make comparisons easier. We do not show the bias of 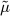 because it is always zero, except for sampling error. All of the shrinkage estimators generally outperform the MLE in terms of bias and 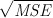, the exception being simulation 15, scenario *IV*. Carreras and Brannath 10 show theoretically that the MLE is maximally biased when all treatment means are equal. The results of scenario *II* supports this. 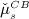 and 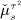
s performances are fairly equal. 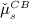 tends to have a smaller bias than 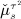 but a larger 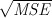. The performance of 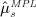 varies considerably; it is the best estimator in terms of 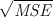 in 8 out of 16 simulations but is sometimes the worst estimator by far (e.g. simulations 11 and 15). Unlike the other shrinkage estimators, it is also negatively biased in general. 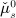 and 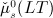 perform similarly and are consistently the most reliable estimators across the 16 scenarios. It is perhaps surprising that their similarity extends to scenarios *III* and *IV*, where one would suspect the LT strategy would come into play. This implies that the difference between 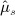 and 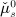 is still almost always less than 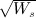.

**Table 1:** Bias and mean squared error (MSE) of the various estimands over the 16 scenarios of a two-stage drop-the-loser trial with *k* = 6 initial treatments.

*σ*_1_,*σ*_2_ values	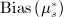						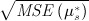						
	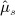	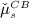	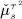	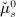	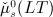	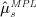	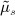	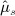	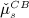	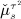	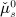	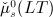	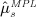
Scenario *I*: true means ∼ *N*(0,1)													
1.(1,1)	0.63	0.19	0.22	0.11	0.11	− 0.17	1.21	1.12	0.97	0.96	0.95	0.94	0.97
2.(2,1)	0.51	0.18	0.29	0.11	0.11	− 0.03	1.08	1.08	0.98	0.97	0.92	0.92	0.91
3. 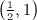	0.51	0.13	0.14	0.11	0.11	− 0.22	1.35	1.08	0.99	0.99	0.98	0.98	1.04
4. 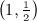	0.40	0.12	0.17	0.00	0.00	− 0.14	1.07	1.05	0.99	0.98	0.98	0.98	1.01
													
Scenario *II*: true means all 0													
5.(1,1)	0.89	0.35	0.45	0.35	0.36	0.16	1.27	1.23	0.92	0.87	0.79	0.79	0.65
6.(2,1)	0.57	0.22	0.39	0.22	0.23	0.12	1.09	1.10	0.97	0.95	0.83	0.83	0.78
7. 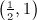	1.14	0.45	0.48	0.45	0.47	0.17	1.64	1.35	0.86	0.84	0.81	0.82	0.58
8. 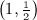	0.57	0.22	0.39	0.23	0.23	0.12	1.08	1.09	0.96	0.94	0.83	0.83	0.77
													
Scenario *III*: one true mean = 1, 5 = 0													
9.(1,1)	0.78	0.25	0.32	0.21	0.22	− 0.03	1.24	1.19	0.94	0.93	0.88	0.88	0.84
10.(2,1)	0.55	0.20	0.36	0.19	0.19	0.08	1.08	1.09	0.97	0.95	0.85	0.85	0.81
11. 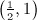	0.59	− 0.07	− 0.06	− 0.10	− 0.09	− 0.56	1.40	1.14	1.04	1.05	1.05	1.04	1.20
12. 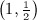	0.50	0.16	0.28	0.11	0.11	− 0.02	1.08	1.08	0.98	0.97	0.93	0.93	0.93
													
Scenario *IV*: one true mean = 1.5, 5 = 0													
13.(1,1)	0.64	0.11	0.16	0.05	0.06	− 0.24	1.21	1.14	0.98	0.99	0.98	0.98	1.02
14.(2,1)	0.53	0.19	0.33	0.16	0.16	0.04	1.08	1.08	0.97	0.96	0.88	0.88	0.86
15. 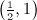	0.21	− 0.40	− 0.39	− 0.43	− 0.43	− 0.94	1.17	1.04	1.16	1.17	1.19	1.18	1.49
16. 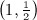	0.40	0.07	0.16	− 0.01	− 0.01	− 0.16	1.07	1.05	0.99	0.99	1.01	1.01	1.04

**Figure 1:**
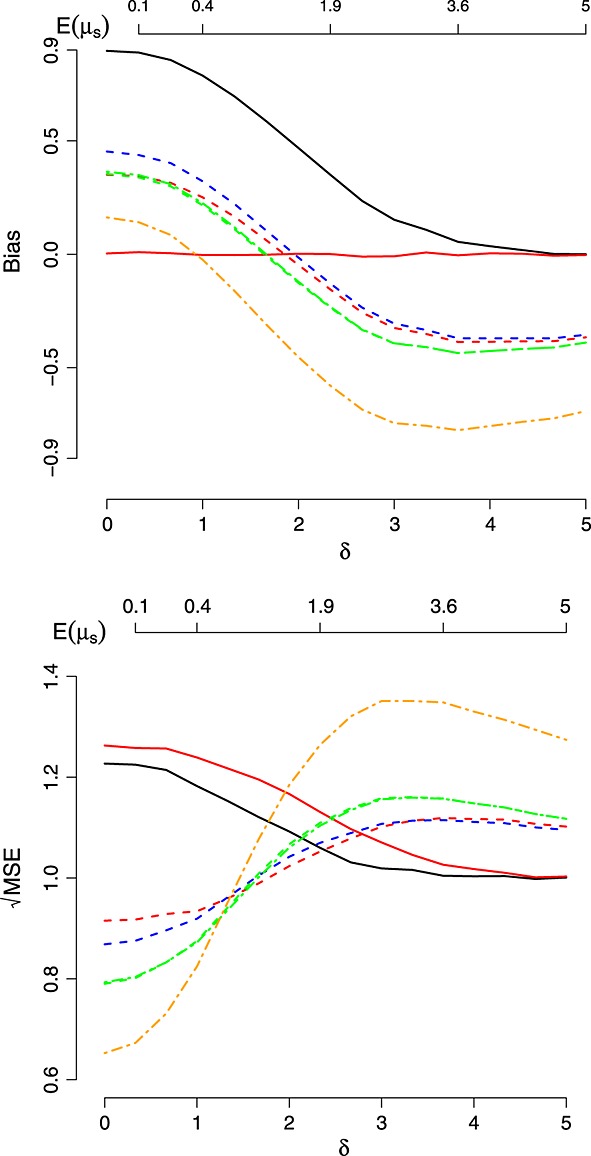
Bias and mean squared error (MSE) of the estimators as a function of *δ*. Key: maximum likelihood estimate (black), UMVCUE (red), 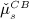 (red-dashed), 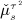 (blue-dashed), 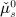 (green dashed), 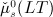 (green dot-dashed) and 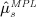 (orange dot-dashed).

Figures 3 show the results of three further simulation studies. In each case, *σ*_1_ = *σ*_2_ = 1. Figure 1 shows the scaled bias and 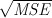 of the estimators for a trial with *k* = 6 treatments, 5 of which have true mean 0 and one of which has true mean *δ*, as *δ* is varied between 0 and 5. In order to highlight the strength of the selection effect as a function of *δ*, we also plot the average value of *μ*_*s*_ (labelled as ‘ *E*[*μ*_*s*_]’). One can see that the bias of the shrinkage estimators changes sign as *δ* increases whereas the bias of the MLE decreases from 0.8 to 0 as *δ* increases. Of the shrinkage estimators, 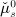 and 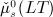 have the smallest bias and MSE (and are indistinguishable) for *δ* up to 1.8. For *δ ≤*2.2, the shrinkage estimators dominate the MLE in terms of 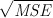. 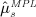 has the smallest bias of all for *δ ≤*1.5 but, by far, has the largest (negative) bias as *δ* increases. 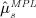 also has the smallest 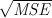 of all estimators for small *δ*, but as *δ* increases, its MSE increases dramatically.

**Figure 2:**
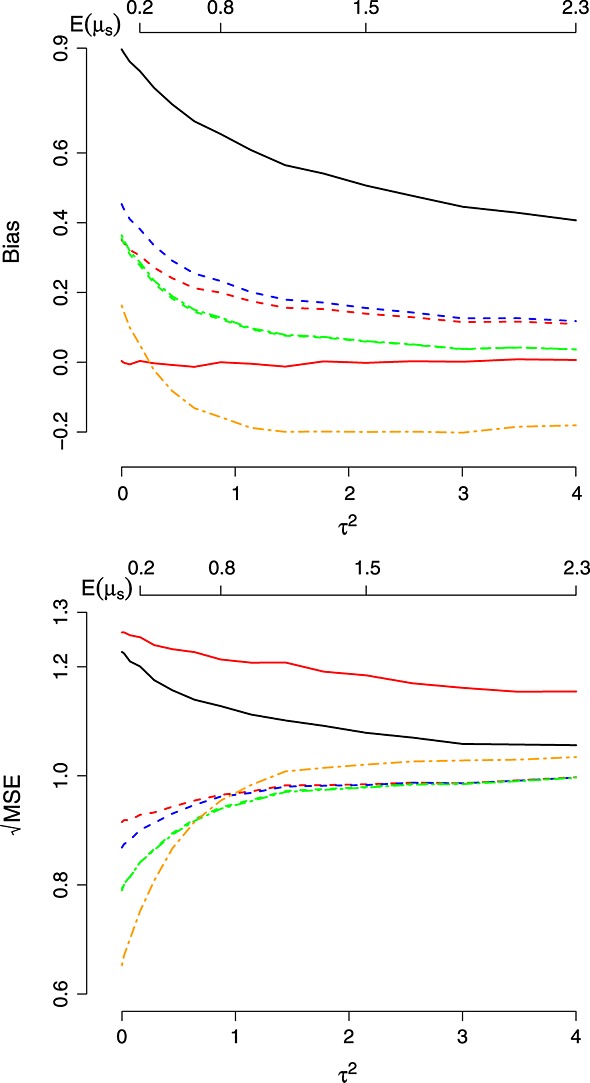
Bias and mean squared error (MSE) of the estimators as a function of *τ*^2^. Key: Same as Figure 1.

**Figure 3:**
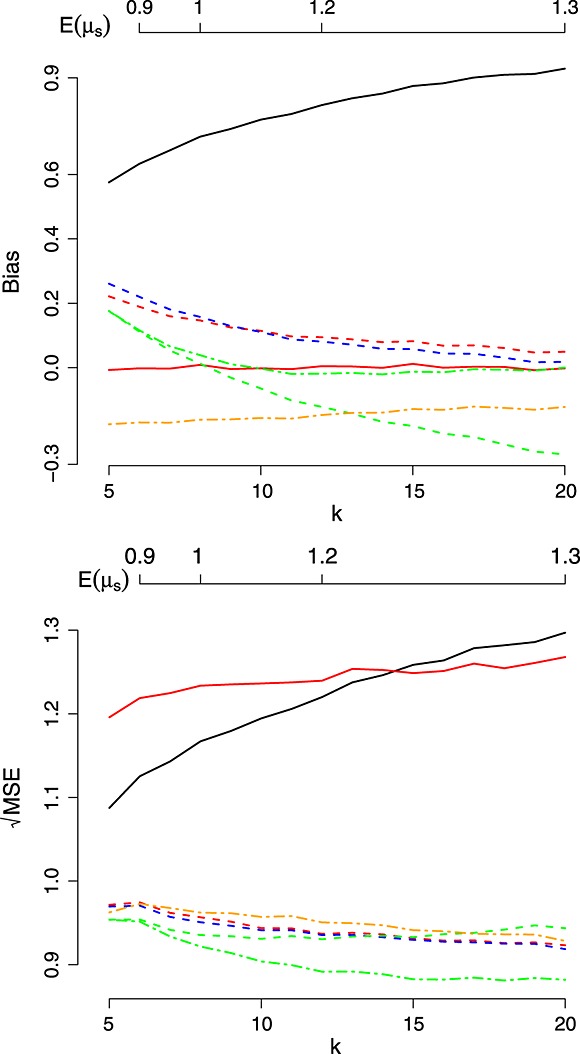
Bias and mean squared error (MSE) of the estimators as a function of *k*. Key: Same as Figure 1.

Figure 2 shows the results of a simulation for *k* = 6 treatments with mean parameters drawn from a *N*(0,*τ*^2^) distribution as *τ*^2^ is varied between 0 and 4 (the choice of *μ* = 0 is clearly unimportant). The bias of all shrinkage estimators decreases towards 0 as *τ*^2^ increases, although this happens most rapidly for 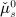 and 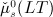 so that they are the least biased. For values of *τ*^2^ greater than 1, 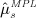 is consistently negatively biased. The 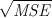 of the shrinkage estimators appears to asymptote upwards towards 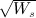 (towards 1 after scaling) as *τ*^2^ increases, but remain below that of the MLE in this range.

In an effort to separate 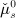 and 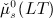, Figure 3 shows the results of a simulation assuming that the treatment mean parameters are drawn from a *N*(0,1) distribution, but the number of treatments, *k*, is varied between 5 and 20. As *k* increases, the positive bias exhibited by 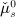 decreases, quickly becoming large and negative. 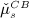 and 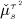 do not to suffer in the same way; their biases asymptote towards 0 as *k* increases. 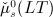 appears to protect 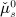 well from its tendency for negative bias beyond *k* = 10. From the right-hand panel, one can see that the price 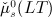 pays for this bias protection is an increase in 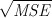. Interestingly, as *k* increases beyond 15, even the UMVCUE has a smaller 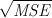 than the MLE.

### 5.1. Summary of findings

Across all simulations, the performance of 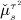 is most similar to Carreras and Brannath's original estimator 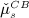, but the two estimators that performed the best were 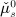 and 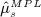. However, the reasons for the latter's apparent success are now qualified. Estimation of the heterogeneity parameter *τ*^2^ is challenging when its true value is small or the number of treatments is small. When *k* is small or there is little apparent variation between treatment effect estimates 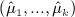, the estimate for *τ*^2^ is often likely to be at or close to zero, even when its true value is larger. This fact is well documented in the meta-analysis literature, where quantification of the (between trial) heterogeneity parameter is often not recommended unless the number of studies is sufficiently large (at least 10). Although poor estimation of *τ*^2^ impacts 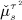 and 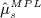, the latter is most strongly affected because from equation (10) when *τ*^2^ = 0, it reduces via equation (11) to the fixed effects estimate for *μ*, 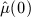. However, when the Paule–Mandel estimate for *τ*^2^ is zero, 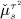 does not shrink to exactly 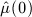 because 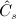 in equation (14) is not equal to 0. This explains why 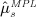 performs so well in Scenario *II*, Table1, and for small values of *δ*,*τ*^2^,*k* in Figures 1– 3 because in these situations, the true value of *μ*_*s*_ is always close (or equal) to *μ*. Conversely, it also explains why it performs badly when *μ*_*s*_ is truly very different from *μ* (e.g. Scenario *IV*, Table1, and large values of *δ*,*τ*^2^,*k* in Figures 1– 3).

The LT version of 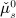 only helped to improve its performance in simulations when the number of treatments rose above 10, which is unrealistically large for most clinical trials settings. It therefore appears to be an unnecessary extension in this context. However, it could potentially be implemented in a more sophisticated manner than we have here. For example, the width of the protection region around the MLE can be tuned to control its bias and MSE, rather than being fixed at a specific value as we did. Efron and Morris 22 provide the theoretical framework for doing this in a general shrinkage estimation context, but their method would need to be altered before application to drop-the-loser designs, in order to account for the selection mechanism. This is a topic for further research. One could also argue that by shrinking the prior variance for *μ*_*s*_ after selection, 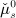 already contains and in-built form of LT.

### 6. Discussion

In this paper, we have reviewed the shrinkage estimation strategy of Carreras and Brannath 10 for two-stage drop-the-loser designs. As an extension, we propose that rather than using only the first stage data, the stage two data should also be used to furnish a single shrinkage equation. Although this strategy replaces two formulae with one, evaluation of this single shrinkage formula is much harder because the variance of its *k* estimated components are no longer equal. By incorporating the methods of Carter and Rolph 16 and Efron and Morris 22, we identified several alternative procedures. The alternative approaches necessitate either explicit estimation of an additional between treatment arm heterogeneity parameter *τ*^2^ (for 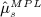 and 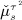) or a different (and quite unnatural) prior distribution for the mean treatment effects (for 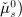. The new methods tend to outperform Carreras and Brannath's original estimator, but unfortunately, no equivalent dominance results could be shown.

From looking at the simulations in totality, the estimator that consistently performs well when *μ*_*s*_ is close to and far from the overall mean of all treatments is 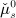. Some may object philosophically to its use in this context because of concerns over Temporal Coherency. However, as Cox 25 states: There may be certain situations where it is perfectly right to modify one's prior beliefs as more data become available. Furthermore, when one's prior uncertainty about a parameter *is* allowed to change over time in a manner proportional to the (increasing) size of the data sample, then the resulting Bayesian inference starts to approximate a classical significance test 26. This is exactly what occurs in the drop-the-loser context when, at the point of selection, the variance of the prior for *μ*_*s*_ shrinks from 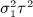 to *W*_*s*_*τ*^2^ – for example, by a factor of 2 when 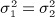. It is therefore pertinent to note that the formula for 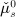 can also be arrived at by applying Lindley's original equal variance shrinkage formula (5) to the standardised MLEs, 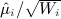, 16 because they are sufficient test statistics for the null hypothesis *μ* = 0.

We chose to illustrate the different estimation approaches for trials involving five or more treatments. Apart from 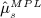, all of the shrinkage estimators discussed in this paper are only defined for 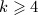, as indicated by the factors of (*k* − 3) they contain. However, we can crudely apply them for the *k* = 3 case by simply replacing these terms with (*k* − 2) instead – as performed by Carreras and Brannath 10. We repeated the simulations shown in Figures (1) and (2) for *k* = 3 using this crude fix to see how it affected the performance of the various estimators. The results (not shown) were qualitatively very similar.

Throughout this paper, we have attempted to stress the link between shrinkage estimation in the adaptive trial context with that of meta-analysis. We have shown that 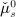, which incorporates the fixed effects estimate 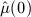, works well as an estimator for *μ*_*s*_ under drop-the-losers selection. It is therefore interesting to note the following: In meta-analyses that exhibit substantial amounts of between study heterogeneity, the random effects estimate for *μ* is known to be unreliable when the heterogeneity is thought to be driven by dissemination bias (i.e. selective reporting and publication of extreme findings) 27,18. In order to address this, it has been advocated that the fixed effects estimate 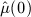 be used as the preferred measure of overall effect instead 28,29. Thus, despite the fact that in the adaptive trial setting, the parameter of interest is 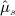 and in meta-analysis, it is the overall grand mean *μ*, when the data are affected by some form of selection, Carter and Rolph's proportional prior approach appears to be an effective solution to both problems.

Bowden and Glimm 30 have extended the idea of a two-stage drop-loser-trial to allow the best performing treatment to be identified over multiple stages. The motivation for adding further stages of selection is that one can markedly increase the probability of selecting the truly best treatment (and subsequently declaring it effective in a confirmatory analysis), whilst keeping trial costs to a minimum. Many other multi-arm multi-stage (MAMS) designs incorporating treatment selection rules have also been proposed with a similar motivation in mind, see for example 31,32. Because they ignore the selection process altogether and use all of the data to define a target posterior distribution or shrinkage equation, 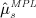, 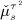 and 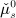 should be simple to apply in any of these contexts. It is not so obvious to see how Carreras and Brannath's original estimator, 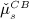, would generalise to the multi-stage context or if the dominance results that make it attractive in the two-stage case would remain in intact.

A simple, straightforward translation of the shrinkage estimators proposed here to other multi-arm trial designs is only immediate if the *k* treatment effect estimates are independent before selection. If, as in the MAMS design of Royston *et al*. 33, the treatment effect summarised time-to-event data in the form of a log-hazard ratio, then the effect estimates would be intrinsically correlated across treatment arms because of their shared control group data. Extending shrinkage estimation to account for inter-dependence of this sort is another topic for further research.

## A Appendix Details for the calculation of

Assume without loss of generality that *s* = 1, so that *μ*_1_ is the mean of the best performing treatment. Let **Z** = (*X*_1_,*Y*_1_,*X*_2_,…,*X*_*k*_,) equal the complete vector of data from the two-stage trial. Under the framework of equation (9), the model induces the unconditional distribution **Z** ∼ N(*μ***1**_*k* + 1_, ***Σ***), where

Assuming that *μ* and *τ*^2^ are known, then the best linear prediction of *μ*_1_ | *μ* is given in equation (10), which is equivalent to

The empirical question is how to estimate *μ* and *τ*^2^ as accurately as possible. If we were to assume that *τ*^2^ is known, then ***Σ*** becomes a completely known matrix. Then, it is easily seen from differentiating ***a* ′ *****Σ*****a** + *λ****a* ′ ****1**_*k* + 1_ with respect to **a** and *λ* that the linear combination ***a* ′ *Z***, which minimises Var(***a* ′ *Z***) = ***a* ′ *****Σ*****a**, is given by

As this also maximises the multivariate normal likelihood, ***a* ′ *Z*** is also equivalent to the MLE for *μ*. Because Σ is diagonal, its inverse is trivially

Hence, we obtain the estimate for  given in equation (11). We then plug  into the log-likelihood for *τ*^2^ given *μ*

and maximise to obtain an estimate for *τ*^2^
